# Flow diverter tail malapposition after implantation in the internal carotid artery for aneurysm treatment: a preliminary study

**DOI:** 10.3389/fneur.2023.1301046

**Published:** 2023-11-23

**Authors:** Zheng Wan, Tianyi Liu, Ning Xu, Wenhao Zhu, Yuan Qi, Chunyang Ma, Hao Chen, Honglei Wang

**Affiliations:** Department of Neurosurgery, The First Hospital of Jilin University, Changchun, China

**Keywords:** flow diverter, intracranial aneurysm, internal carotid artery, malapposition, risk factors

## Abstract

**Background and purpose:**

Favorable wall apposition of a flow diverter (FD) is essential for the treatment of intracranial aneurysms. The irretrievability and final drop point uncertainty of the proximal tail of the FD increase the difficulty of achieving good tail apposition. Therefore, understanding the factors associated with FD tail malapposition would be helpful for clinical practice.

**Methods:**

A total of 153 patients with 161 FD deployments in the carotid artery between 2020 and 2023 were retrospectively collected from our center’s database for this study. Patient demographics, aneurysm characteristics, FDs, carotid artery anatomy, periprocedural complications, discharge modified Rankin scale (MRS) scores, and follow-up outcomes were investigated by comparing patients with and without FD tail malapposition. Comparisons were made with t tests or Kruskal–Wallis tests for continuous variables and the Pearson χ^2^ or Fisher exact test for categorical variables. Logistic regression was conducted to determine the predictors of malapposition.

**Results:**

Tail malapposition occurred for 41 out of the 161 FDs (25.5%). Univariate analysis revealed that the FD brand, FD length, FD distal to proximal vessel diameter ratio, FD tail position (straight or curved), and curvature of the vessel curve were significantly associated with FD tail malapposition (*p* < 0.05). Further multivariate analysis demonstrated that the application of a surpass FD (*p* = 0.04), the FD distal to proximal vessel diameter ratio (*p* = 0.022), the FD tail position (straight or curved) (*p* < 0.001) and the curvature of the vessel curve (*p* < 0.001) were factors significantly associated with FD tail malapposition. No significant difference was found in periprocedural or follow-up outcomes. The classification of FD tail malapposition was determined from imaging. The two major patterns of FD tail malapposition are unattached tails and protrusive tails.

**Conclusion:**

FD tail malapposition might be associated with a larger FD distal to the proximal vessel diameter difference, a curved vessel where the FD tail is located, and a larger curvature of the vessel curve. FD tail malapposition can be classified into unattached tails and protrusive tails, which have their own characteristics and should be noted in clinical practice.

## 1 Introduction

With the revolutionary vision for the therapeutic strategy of intracranial aneurysms and more clinical evidence, flow diverters (FDs) have been widely applied and commonly accepted in recent years (1–5). However, the risk of procedure-related morbidity and mortality is not negligible (6). One of the key factors guaranteeing the efficacy and safety of FD application is favorable vascular wall apposition. FD malapposition might not only lower the aneurysm occlusion rate (7, 8) but also increase the risk of thrombosis or in-stent stenosis (9, 10).

Compared with the distal and middle parts of an FD, for the proximal tail, it is more difficult to control the course of release to achieve good wall apposition. On the one hand, the retrievability of an FD usually does not include the proximal tail. When the manipulation is not satisfactory, the proximal tail of an FD cannot be adjusted by retrieving and releasing it again. On the other hand, the actual drop point of the FD tail is difficult to predict because of the retraction of the stent itself or the use of the pushing technique during its release, ensuring favorable apposition of the distal and middle parts of the FD. Thus, it is likely that the FD tail is eventually released at the curved vessel. A previous study showed that the proximal part of an FD is the most common segment of FD malapposition, accounting for approximately 59.1% of cases, while the distal and middle parts account for 13.6 and 27.3% of cases, respectively (11).

Although the experiences of expert interventional therapists and FD manufacturers indicate that the proximal tail of the FD should not be released at the vessel curve in cases of malapposition, there has been no relevant research verifying this issue and determining the risk of malapposition when the FD tail drops at the curved vessel. Probable factors associated with FD tail malapposition are still unclear. This study was performed to identify factors related to the occurrence of FD tail malapposition and explore FD tail malapposition patterns in the internal carotid artery.

## 2 Materials and methods

### 2.1 Enrollment

Patients with intracranial aneurysms treated by FDs in our hospital from January 2020 to July 2023 were retrospectively enrolled. The authorized local ethical committee approved this study. Patients were considered for enrolment according to the following: (1) they had aneurysms in the internal carotid artery, (2) they had only one FD deployed within one internal carotid artery, and (3) they had available demographic data, treatment information, and procedural images of distal subtraction angiography (DSA). Patients were not eligible for enrolment according to the following criteria: (1) ruptured aneurysms; (2) more than one FD was deployed within one internal carotid artery and the FDs overlapped; (3) the proximal tail of the FD was not visible or blurred because of a bone artifact or coil block; (4) malapposition of the distal or middle part of the FD was detected; (5) a stent was deployed during the previous treatment; and (6) a Tubridge FD (Microport Medical Company, Shanghai, China) was used for treatment. The Tubridge FD has two platinum-iridium wires for photographic display in DSA, resulting in relative uncertainty of FD tail malapposition occurrence in comparison with other FDs for which the whole body is visible.

### 2.2 Data collection

Data on patient demographics (age and sex), internal carotid artery aneurysm location/side/size, the FD brand and size (diameter and length), coil usage during the operation, procedural complications, and discharge MRS scores were collected from the medical record database of our hospital. Information on the FD tail apposition condition, carotid artery anatomy, and follow-up outcomes (the aneurysm occlusion rate and parent artery changes) was collected from the procedural imaging database of our hospital. Aneurysm obliteration at follow-up was evaluated using the O’Kelly–Marotta grading scale (12): A, at least 95% of the aneurysm remained; B, 5–95% remained; C, <5% remained; and D, complete obliteration. The stasis grade is determined by the timing of the contrast medium clearance from the aneurysm as defined by the phases of the angiogram: 1, no stasis (clearance within the arterial phase, prior to the capillary phase); 2, moderate stasis (clearance prior to the venous phase); 3, significant stasis (contrast persists in aneurysm into the venous phase and beyond); NA, the contrast stasis of aneurysm was blurred and could not be determined to grade 1, 2, or 3 because of the block of the coil while the occlusion rate of aneurysm could be recognized by subtracted 3D rotation imaging.

**Figure 1:**
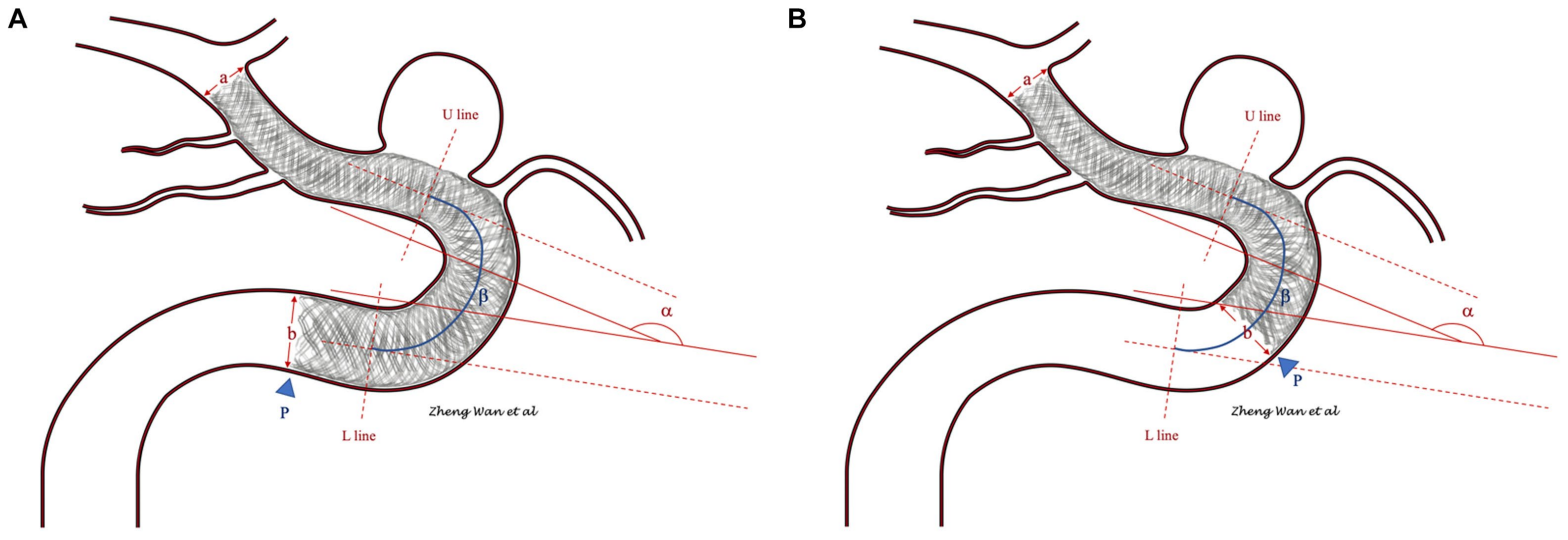
**(A,B)** Show the measuring methods of the carotid artery anatomy parameters. The diameter ratio of the distal and proximal vessels in which the flow diverter was located was measured as the ratio of vessel diameters a and b. The drop position of the flow diverter proximal end was determined by the position of the tail end (blue arrowhead). The segment between the U line and L line was defined as the curved part, and segments above the U line or below the L line and those not included in other curved parts were defined as straight lines. The angle of the flow diverter tail-located vessel was measured as α. The curvature of the flow diverter tail-located vessel was measured as the ratio of α and the length of the curved part β. If the flow diverter tail was located at the straight part of the vessel, the distally curved part of the vessel was included in the angle and curvature statistics.

Malapposition of the FD proximal tail was determined by the observation of unsubtracted images or Vaso CT after implantation. Different patterns of FD tail malapposition were recorded for further evaluation. The carotid artery anatomy information included the diameter ratio of the distal and proximal vessels where the FD was located, the dropping position of the proximal ends of the FD (straight part or curved part of the vessel), turning angle and curvature of the vessel located at the tail of the FD (if the tail of the FD was located at the straight part of vessel, the last distal curved part of the vessel was included in the angle and curvature statistics). The carotid artery anatomy parameters are defined in Figure 1.

### 2.3 Endovascular procedure

Dual antiplatelet medication consisting of aspirin 100 mg/day and clopidogrel 75 mg/day was administered to patients for at least 5 days. Routine preoperative platelet function tests were performed. In patients with clopidogrel resistance, ticagrelor 90 mg/day was administered in place of clopidogrel for a better antiplatelet effect. All FD implantations were performed under general anaesthesia via a femoral approach. According to the characteristics of the parent artery and aneurysm, we selected an appropriate FD size and determined whether to use coils when the size of the aneurysm was larger than 10 mm or the aneurysm had an irregular morphology. During the release of the FD, the pushing technique was used to guarantee favorable wall apposition, and the stent was retrieved and implanted again if the position or apposition of the FD was unsatisfactory. After the implantation of the FD, we evaluated the angiograms to determine whether malapposition had occurred. Methods including manipulation with a microwire, a microcatheter, or an intermediate catheter and balloon angioplasty were used to resolve malapposition. After the operation, the patients were prescribed dual antiplatelet therapy for 6 months, with aspirin being continued indefinitely thereafter. Clinical follow-ups were conducted by DSA.

### 2.4 Statistical analysis

Two neurointerventional specialists separately assessed the DSA images for the recognition of FD tail malapposition. The κ coefficient was calculated for interrater agreement. Continuous variables are presented as the means with SDs, and discrete variables are presented as numbers with percentages (%). Statistical analyses were performed with SPSS 26.0 (IBM Inc., Chicago, Illinois, United States). For univariate analysis, t tests or Kruskal–Wallis tests for continuous variables and the Pearson χ^2^ or Fisher exact test for categorical variables were utilized. For multivariate analysis, variables with *p* values less than 0.2 in univariate analysis that were found to be significant were entered into the logistic regression analysis to further identify independent factors associated with FD tail malapposition. *p* < 0.05 (two-sided) was the criterion for statistical significance.

## 3 Results

A total of 153 patients (mean age: 55.57 ± 9.482; 130 women, 80.4%) with 161 FD deployments in the carotid artery were included in this study. Forty-one out of the 161 FDs (25.5%) were detected to have FD proximal tail malapposition. The interrater agreement with respect to FD tail malapposition was 0.935 (95% CI: 0.872–0.998). The patient demographics, aneurysm characteristics, FD, carotid artery anatomy, periprocedural complications, and discharge MRS scores of the cohort are presented in Table 1.

**Table 1:** Patient, aneurysm, flow diverter, carotid artery anatomy, periprocedural complication and discharge MRS characteristics.

	Tail with good apposition (*n* = 120)	Tail with malapposition (*n* = 41)	*P*-value
Age (years)	55.13 ± 9.192	56.85 ± 10.297	0.317
Female	96 (80%)	34 (82.9%)	0.682
Aneurysm location			0.247
Anterior choroidal artery	5 (4.2%)	2 (4.9%)	
Posterior communicating artery	29 (24.2%)	6 (14.6%)	
Supraclinoid	28 (23.3%)	10 (24.4%)	
Paraopthalmic	38 (31.7%)	19 (46.3%)	
Paraclinoid	6 (5%)	3 (7.3%)	
Cavernous	14 (11.7%)	1 (2.4%)	
Aneurysm side (left)	68 (56.7%)	20 (48.8%)	0.468
Aneurysm size			0.324
<5 mm	51 (42.9%)	23 (56.1%)	
5–10 mm	42 (35.3%)	11 (26.8%)	
10–20 mm	20 (16.8%)	7 (17.1%)	
>20 mm	6 (5%)	0 (0%)	
FD brand			0.030
Pipeline	109 (77.3%)	32 (22.7%)	
Surpass	5 (41.7%)	7 (58.3%)	
Nuva	6 (75.0%)	2 (25.0%)	
FD diameter	4.03 ± 0.46	4.11 ± 0.45	0.378
FD length	21.77 ± 5 0.25	19.34 ± 3.48	0.003
Coil usage	42 (35%)	15 (36.6%)	0.855
DP vessel diameter ratio	0.812 ± 0.103	0.756 ± 0.141	0.023
Position of FD tail			<0.001
Straight part	99 (82.5%)	9 (22.0%)	
Curved part	21 (17.5%)	32 (78.0%)	
Angle of tail vessel curve	119.93 ± 37.93	129.10 ± 36.45	0.147
Curvature of tail vessel curve	11.95 ± 3.91	15.78 ± 4.08	<0.001
Periprocedural complication			0.847
None	115 (95.8%)	39 (95.1%)	
Ischaemia	5 (4.2%)	2 (4.9%)	
Discharge MRS score			0.536
0	117 (97.5%)	39 (95.1%)	
1	1 (0.8%)	2 (4.9%)	
2	1 (0.8%)	0 (0%)	
3	1 (0.8%)	0 (0%)	

### 3.1 Characteristics of patients, aneurysms, FDs, and coil usage

The results are shown in Figure 1. The age, sex, and left or right localization of aneurysms were not significantly different between the two groups of patients with and without FD tail malapposition. For aneurysm location, paraophthalmic aneurysms were the most common in both groups, with a 38% overall occurrence rate. There was no significant difference in the distribution of aneurysm locations between the groups (*p* = 0.247). For aneurysm size, 4 intervals were set: <5 mm, 5–10 mm, 10–20 mm, and > 20 mm. FDs were most frequently used for aneurysms less than 5 mm in both groups, with a 46% overall occurrence rate. There was no significant difference in aneurysm size between the two groups (*p* = 0.324). Three FD brands consisting of 141 pipeline flex stents (Covidien Neurovascular, Dublin, Ireland), 12 Surpass streamline stents (Stryker Neurovascular, Fremont, California) and 8 Nuva stents (Taijieweiye, Beijing, China) were used in the 161 cases. A total of 109 out of the 141 pipeline FDs (77.3%) demonstrated good tail apposition, and 32 (22.7%) pipeline FDs demonstrated tail malapposition. Five out of 12 surpass FDs (41.7%) demonstrated good tail apposition, and 7 (58.3%) surpass FDs demonstrated tail malapposition. Six out of the 8 Nuva FDs (75%) demonstrated good tail apposition, and 2 (25%) demonstrated tail malapposition. Considering the influence of different FD brands on tail malapposition, a statistically significant difference was found (*p* = 0.03) between the two groups. The average diameters of the FDs in the good apposition and malapposition groups were 4.03 ± 0.46 mm and 4.11 ± 0.45 (*p* = 0.378), respectively. The average lengths of the FDs in the good apposition and malapposition groups were 21.77 ± 5.25 and 19.34 ± 3.48, respectively, with a statistically significant difference (*p* = 0.003). For 42 out of the 120 patients (35%) in the good apposition group, coils were concurrently used to embolize the aneurysm; coils were used for 15 out of the 41 patients (36.6%) in the malapposition group (*p* = 0.855).

### 3.2 Characteristics of carotid anatomy

The diameter ratio of distal and proximal vessels in which the FD was located was 0.812 ± 0.103 and 0.756 ± 0.141, respectively, in the good apposition and malapposition groups, with a statistically significant difference (*p* = 0.023). In the good apposition group, 99 out of the 120 FD proximal ends (82.5%) were located at the straight part of the vessel, and 21 (17.5%) were located at the curved part of the vessel. In the FD tail malapposition group, 9 out of the 41 FD proximal ends (22%) were located at the straight part of the vessel, and 32 (78%) were located at the curved part of the vessel. A statistically significant difference (*p* < 0.001) was found for the dropping position of the proximal ends of the FDs. The angles of the tail vessel curve of the good apposition and malapposition groups were 119.93 ± 37.93° and 129.10 ± 36.45°, respectively. There was no statistically significant difference (*p* = 0.147). The curvatures of the tail vessel curve in the good apposition and malapposition groups were 11.95 ± 3.91 and 15.78 ± 4.08, respectively. A statistically significant difference was found (*p* < 0.001). All results are summarized in Figure 1.

### 3.3 Multivariable analysis

Multiple logistic regression was performed to identify the independent factors associated with FD proximal tail malapposition using an ENTER process. The FD brand, FD length, diameter ratio of the distal and proximal vessels, position of the FD tail, angle of the tail vessel curve, and curvature of the tail vessel curve were included for analysis. The results are shown in Table 2. The use of a surpass stent (*p* = 0.04), the diameter ratio of distal and proximal vessels (*p* = 0.022), the position of the FD tail (*p* < 0.001), and the curvature of the tail vessel curve (*p* < 0.001) were independently associated with FD tail malapposition.

**Table 2:** Factors associated with FD tail malapposition in the treatment of carotid aneurysms.

	Odds ratio (95% CI)	*P-*value
FD brand		
Pipeline	1 [Reference]	
Surpass	5.219 (1.08–25.16)	0.040
Nuva	1.96 (0.27–14.36)	0.508
FD length	0.90 (0.79–1.03)	0.143
DP vessel diameter ratio	0.004 (0–0.44)	0.022
Position of the FD tail	19.63 (6.49–59.33)	<0.001
Angle of the tail vessel curve	0.989 (0.97–1.01)	0.195
Curvature of the tail vessel curve	1.414 (1.18–1.70)	<0.001

### 3.4 Periprocedural and follow-up outcomes

Seven out of 153 patients (4.6%) experienced periprocedural ischemic events. No hemorrhagic events were observed in any of the patients. The periprocedural complication rates of the good apposition and malapposition groups were 4.2 and 4.9%, respectively. No statistically significant difference was found (*p* = 0.847). The discharge MRS score was 0 for 117 patients (97.5%), 1 for 1 patient (0.8%), 2 for 1 patient (0.8%), and 3 for 1 patient (0.8%) in the good apposition group. The discharge MRS score was 0 for 39 patients (95.1%) and 1 for 2 patients (4.9%) in the malapposition group. No statistically significant difference was found (*p* = 0.536). The results of periprocedural complications are shown in Table 1.

The average follow-up time intervals of the good apposition and malapposition groups were 9.66 ± 4.424 months and 10.58 ± 5.470 months, respectively (*p* = 0.571). There was no statistically significant difference was found between the occlusion rate and contrast stasis of aneurysms in the good apposition group and the malapposition group (*p* = 0.114). Three out of 56 patients (5.4%) showed stenosis of the carotid artery in the good apposition group, and 3 out of 19 patients (15.8%) showed stenosis of the carotid artery in the malapposition group (*p* = 0.166). The results of the follow-up outcomes are summarized in Table 3.

**Table 3:** Characteristics of the aneurysm occlusion rate and parent artery of the FD during follow-up.

	Tail with good apposition (*n* = 56)	Tail with malapposition (*n* = 19)	*P*-value
Follow-up time interval	9.66 ± 4.424	10.58 ± 5.470	0.571
Aneurysm occlusion rate			0.114
A	0 (0%)	0 (0%)	
A1	0	0	
A2	0	0	
A3	0	0	
B	12 (21.4%)	1 (5.3%)	
B1	2	0	
B2	1	1	
B3	8	0	
NA	2	0	
C	10 (17.9%)	3 (15.8%)	
C1	0	1	
C2	2	1	
C3	4	0	
NA	4	1	
D	34 (60.7%)	15 (78.9%)	
Parent artery of FD			0.166
Normal	53 (94.6%)	16 (84.2%)	
Stenosis	3 (5.4%)	3 (15.8%)	

### 3.5 Patterns of FD tail malapposition and their peculiar characteristics

**Figure 2:**
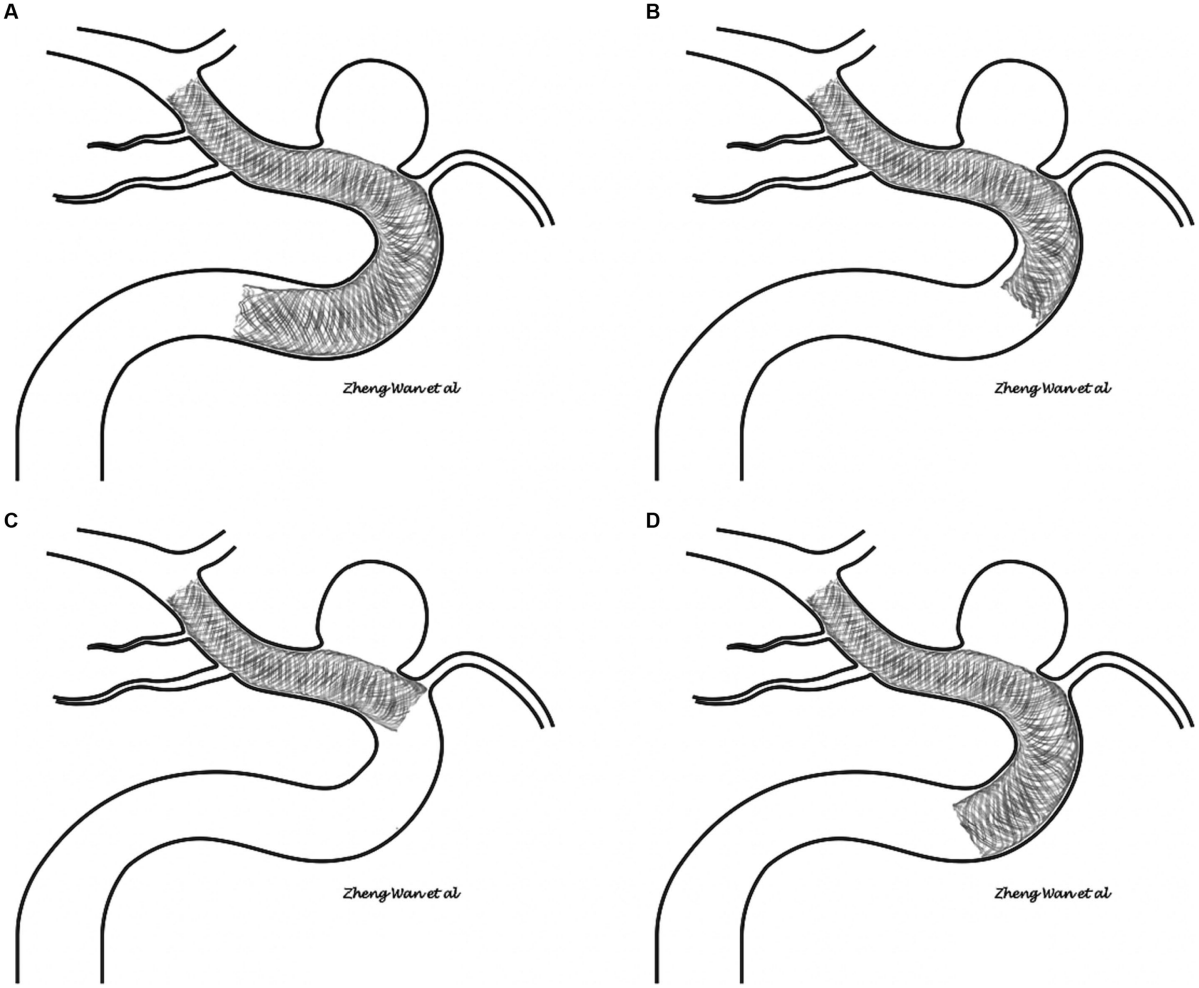
**(A,B)** Sketch of the unattached tail pattern of flow diverter tail malapposition. **(C,D)** Sketch of the protrusive tail pattern of flow diverter tail malapposition.

**Figure 3:**
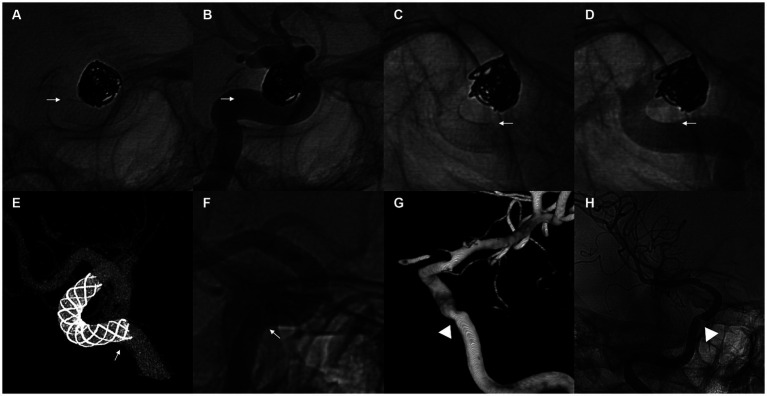
**(A–D)** Demonstration of a representative case of an unattached tail pattern of flow diverter tail malapposition. A 68-year-old woman with a supraclinoid aneurysm underwent flow diverter implantation using a 4 mm × 18 mm Pipeline Flex. The proximal tail of the stent was unattached to the vessel wall of the less curved side after complete release **(A,B)**. Manipulation with a J- or S-shaped microwire and microcatheter was applied, and the malapposition of the proximal tail of the stent was resolved **(C,D)**. White arrows point to the less curved side of the stent. **(E,F)** Demonstrate a representative case of a protrusive tail pattern of flow diverter tail malapposition. A 40-year-old woman with a paraophthalmic aneurysm underwent flow diverter implantation using a 5 mm × 18 mm Nuva stent. The proximal tail of the stent was protrusive at the curved vessel, and incomplete apposition of the less curved side of the stent was observed **(E,F)**. White arrows point to the position of the protrusive tail. Follow-up outcomes at 6 months showed that the aneurysm was completely occluded, but moderate stenosis occurred at the position of the protrusive tail **(G,H)**. White arrowheads point to the position of stenosis at follow-up.

After a thorough and systematic review of the imaging of all FD proximal tail malapposition cases, two major patterns of FD tail malapposition were recognized: unattached tails and protrusive tails. A sketch of the two patterns is shown in Figure 2, and representative cases are shown in Figure 3. Out of the 41 cases of FD tail malapposition, 17 demonstrated tail malapposition that was unattached to the vascular wall of the less curved side, 1 case demonstrated tail unattachment to the vascular wall of the greater curved side, 22 cases demonstrated tail protrusion at the vascular turning corner leading to malapposition on the less curved side, and 1 case demonstrated concurrent tail unattachment to the vascular wall of the less curved side and protrusive tail. The only case with an unattached tail of the greater curved side occurred because of atherosclerotic sag at the greater curved side of the tail-located vessel (Figure 3). Therefore, this pattern was not classified as a common pattern of FD tail malapposition. We further compared the characteristics of the cases of unattached tail and protrusive tail, and the results are shown in Table 4. There was no statistically significant difference in patient age, sex, aneurysm location/side/size, FD brand/diameter/length, coil usage, angle and curvature of the tail vessel curve, periprocedural complications or discharge MRS scores between the unattached tail group and protrusive tail group. The diameter ratio of distal and proximal vessels in the unattached tail group was significantly lower than that in the protrusive tail group (*p* = 0.048). Fifty percent of cases occurred at the curved part of the vessel in the unattached tail group, and 100% of cases occurred at the curved part of the vessel in the protrusive tail group (*p* < 0.001). The rate of remedy implementation in the unattached tail group was significantly higher than that in the protrusive tail group (*p* = 0.022). The successful remedy treatment rates of the unattached tail and protrusive tail groups were 80 and 40%, respectively. No statistically significant difference was found (*p* = 0.087).

**Table 4:** Comparison between unattached tails and protrusive tails.

	Unattached tails (*n* = 18)	Protrusive tails (*n* = 22)	*P-*value
Age (years)	60.44 ± 7.065	54.95 ± 11.039	0.076
Female sex	13 (72.2%)	20 (90.9%)	0.211
Aneurysm location			0.988
Anterior choroidal artery	1 (5.6%)	1 (4.5%)	
Posterior communicating artery	2 (11.1%)	3 (13.6%)	
Supraclinoid	4 (22.2%)	6 (27.3%)	
Paraopthalmic	9 (50%)	10 (45.5%)	
Paraclinoid	2 (11.1%)	1 (4.5%)	
Cavernous	0 (0%)	1 (4.5%)	
Aneurysm side (left)	10 (55.6%)	9 (40.9%)	0.525
Aneurysm size			0.326
<5 mm	9 (50%)	13 (59.1%)	
5–10 mm	7 (38.9%)	4 (18.2%)	
10–20 mm	2 (11.1%)	5 (22.7%)	
>20 mm	0 (0%)	0 (0%)	
FD brand			0.413
Pipeline	16 (90.8%)	16 (78.0%)	
Surpass	1 (4.2%)	5 (17.1%)	
Nuva	1 (5.0%)	1 (4.9%)	
FD diameter	4.24 ± 0.47	4.01 ± 0.45	0.125
FD length	20.44 ± 4 0.25	18.65 ± 2.46	0.24
Coil usage	8 (44.4%)	7 (31.8%)	0.517
DP vessel diameter ratio	0.705 ± 0.132	0.793 ± 0.140	0.048
Position of the FD tail			<0.001
Straight part	9 (50%)	0 (0%)	
Curved part	9 (50%)	22 (100%)	
Angle of the tail vessel curve	138.61 ± 29.096	119.86 ± 40.368	0.107
Curvature of the tail vessel curve	15.20 ± 3.42	16.13 ± 4.64	0.485
Periprocedural complications			0.196
None	16 (88.9%)	22 (100%)	
Ischemia	2 (11.1%)	0(0%)	
Discharge MRS score			0.196
0	16 (88.9%)	22 (100%)	
1	2 (11.1%)	0 (0%)	
Remedy implementation	15 (83.3%)	10 (45.5%)	0.022
Successful rate of remedy	12 (80%)	4 (40%)	0.087

The average follow-up time intervals of the unattached tail and protrusive tail groups were 10.60 ± 4.195 months and 10.56 ± 6.894 months, respectively (*p* = 0.65). There was no statistically significant difference in the aneurysm occlusion rate, aneurysm contrast stasis, or vessel changes during follow-up (*p* = 1, *p* = 0.582) as shown in Table 5.

**Table 5:** Comparison between unattached tails and protrusive tails during follow-up.

	Unattached tails (*n* = 10)	Protrusive tails (*n* = 9)	*P-*value
Follow-up time interval	10.60 ± 4.195	10.56 ± 6.894	0.65
Aneurysm occlusion rate			1
A	0 (0%)	0 (0%)	
A1	0	0	
A2	0	0	
A3	0	0	
B	1 (10%)	0 (0%)	
B1	0	0	
B2	1	0	
B3	0	0	
C	2 (20%)	1 (11.1%)	
C1	1	0	
C2	0	1	
C3	0	0	
NA	1	0	
D	7 (70%)	8 (88.9%)	
Parent artery of FD			0.582
Normal	9 (90%)	7 (77.8%)	
Stenosis	1 (10%)	2 (22.2%)	

## 4 Discussion

FDs are stents with high porosity and high metal surface coverage that provide a novel therapeutic strategy for intracranial aneurysms. However, most likely because of previous studies about the incomplete apposition of the common stents used in aneurysm coil embolization (13–15), studies aimed at FD tail malapposition are scarce. Considering the design and structure of FDs, favorable wall apposition should be more important for the safety and efficacy of treatment and has its own characteristics. As previously mentioned, it is more difficult to guarantee favorable wall apposition in the proximal tail of the FD because of the irretrievability and uncertainty of the final drop point. Wang et al. (11) conducted the first and only study of FD malapposition by separating the stent into distal, middle and proximal parts. The proximal part of the FD had the highest rate of malapposition, corresponding to its characteristics of release.

In the present study, we reported that 25.5% of patients with FD implants had radiographically identifiable proximal tail malapposition. This rate is higher than that of FD tail malapposition (11%) in the study by Wang et al. (11). We speculate that the increased attention and importance given to the malapposition pattern of the protrusive tail contribute to the discrepancy. The rate of unattached tail malapposition was 11.2% (18 out of 161) in our study, which is similar to that of 11% in the study by Wang et al. (11).

The tortuous running of the parent artery is deemed to be associated with the incomplete apposition of stents (15, 16). In this study, the diameter ratio of the distal and proximal vessels, the position of the FD tail, and the curvature of the tail vessel curve were independent risk factors associated with FD tail malapposition. The results reveal that a discrepancy in the vessel diameter at which the FD is located distally and proximally, an FD proximal end dropping at the curve part and a larger curvature can increase the risk of FD tail malapposition. Kato et al. (17) conducted research on the factors associated with FD malapposition in the treatment of large and giant aneurysms with a sample size of 26 patients. They also measured the proximal and distal vessel diameter discrepancy and siphon angle for analysis. However, no statistically significant difference was found in these factors. Interestingly, they found that relative malapposition increased by an estimated 13% (95% confidence interval: 4–23%, *p* = 0.006) for every 1-mm increase in the FD diameter, whereas the FD diameter was not associated with malapposition (*p* = 0.378) in our study. The discrepancy in the results might come from the difference in the studied aneurysms (all-size aneurysms in our study versus >10 mm aneurysms in their study), the studied FD part (FD proximal tail in our study versus the whole stent in their study) and the sample size. The angle of the vessel curve was not associated with FD malapposition in our study or the study by Kato et al. (17). However, we determined that the curvature of the vessel curve was statistically related to FD malapposition. This result indicated that only considering the angle of the vessel curve that the FD passes through is not enough for malapposition prediction. The degree of vessel curve turning rather than the angle is the factor that influences the apposition of an FD.

In this article, we first classified FD proximal tail malapposition into two major patterns: unattached tails and protrusive tails. Unattached tails are a commonly recognized malapposition pattern, while protrusive tails have received little attention. However, a protrusive tail has already been depicted in a few studies without differentiation from the pattern of an unattached tail (17–19). Kühn et al. (18) described a case of protrusive tail malapposition and implanted an open-cell stent for treatment. In fact, most protrusive tail FDs are usually overlooked except for obvious FDs. It is possible for a protrusive tail to occur when the proximal end of the FD is dropped at the vessel curve. It seems that an unattached tail is more relevant to the proximal and distal vessel diameter difference than a protrusive tail. With respect to remedy implementation, more than half of the protrusive tail patients did not receive any treatment. The success rate of remedy of the protrusive tail is lower than that of the unattached tail. The protrusive tail is often unable to be resolved by manipulation with a microwire, a microcatheter, or an intermediate catheter and balloon angioplasty. Only shortening of the FD tail achieved by conducting these manipulations can resolve the issue, whereas the FD is usually contracted enough during release by the pushing technique and is harder to contract after full release. Implanting another stent might be effective, but we did not use this strategy in our patients. The results remind us to avoid the release of the proximal FD in the curved vessel to reduce the risk of incomplete apposition.

Surpass FD implantation is also related to FD tail malapposition. A total of 87.3% of surpass tail malapposition is protrusive tail pattern, while the rate of unattached tail pattern of surpass is similar to that of other FD brands. However, the number of Surpass and Nuva FDs in this study was much lower than the number of Pipeline FDs and did not have enough efficacy for precise statistical prediction. The result might come from quantitative bias. The results of our study indicate a probable incline that surpass FD implantation might be associated with the occurrence of a protrusive tail. This probably results from the characteristics of the Surpass FD structure. Compared to the Pipeline and Nuva stents, Surpass stents have more metal strings constructing the stent, leading to more stiffness. Therefore, a stiffer trait makes it harder to conform with the running of the vessel at the curved part and makes it easier for malapposition of the protrusive tail to occur. Therefore, more studies about this issue are warranted.

Although it is recognized that incomplete apposition of FD is associated with more thromboembolic events, our results did not show a statistically significant difference between the good apposition and malapposition groups during the periprocedural period and follow-up. This result might be limited by the sample size and strict administration of dual antiplatelet therapy. However, stenosis of the parent artery related to the FD tail occurred in 15.8% of the patients in the malapposition group and 5.4% of the patients in the good apposition group. The higher rate of stenosis might result from the blood flow disturbance caused by FD tail malapposition. Although there was no early follow-up (<6 months), the proximal parent artery sometimes had excessive stimulation owing to the remedy process of tail malapposition with manipulation with a microwire, a microcatheter, or an intermediate catheter, thus resulting in a higher occurrence rate of vasospasm. This might increase the risk of delayed ischemic stroke (20). Only one case of near-occluded internal carotid artery was found at follow-up and was in the malapposition group. Furthermore, there was no significant difference in the aneurysm occlusion rate at follow-up between the two groups. This result might indicate that even if the proximal part of the FD is incompletely adhered to the vessel wall, a good apposition of the part of the FD surrounding the aneurysm can exert a diverting effect to occlude the aneurysm.

This study has several shortcomings. Firstly, although we included different brands of FD as their design, manufacturing process, and behavior differ considerably, there were not enough Surpass and Nuva cases for favorable power of statistical analysis. Secondly, the evidence for the follow-up changes of aneurysm occlusion and parent artery resulting from the FD tail malapposition was still scarce because of the limited number of follow-up cases. Thirdly, multiple statistical testing without correction might give rise to a bias for statistical comparison.

## 5 Conclusion

In this retrospective study, the incidence and related factors of FD proximal tail malapposition were explored. The results showed that a larger FD distal to the proximal vessel diameter difference, a curved FD tail position, and a larger curvature of the vessel curve were associated with FD proximal tail malapposition. Unattached tails and protrusive tails are two major patterns of FD tail malapposition with their own characteristics and should be noted in clinical practice. Whether different brands of FD with different structure designs are associated with the occurrence of FD tail malapposition still needs more studies to verify. Prudently selecting an appropriately sized FD and careful manipulation during the procedure to make the proximal end drop at the straight vessel is crucial to decreasing the incidence of FD tail malapposition.

## Data availability statement

The raw data supporting the conclusions of this article will be made available by the authors, without undue reservation.

## Ethics statement

The studies involving humans were approved by the Ethics Committee of the First Hospital of Jilin University. The studies were conducted in accordance with the local legislation and institutional requirements. Written informed consent from the patients/participants or patients/participants’ legal guardian/next of kin was not required to participate in this study in accordance with the national legislation and the institutional requirements.

## Author contributions

ZW: Project administration, Writing – original draft. TL: Formal analysis, Writing – review & editing. NX: Methodology, Writing – review & editing. WZ: Validation, Writing – review & editing. YQ: Writing – review & editing. CM: Data curation, Writing – review & editing. HC: Data curation, Writing – review & editing. HW: Conceptualization, Supervision, Writing – review & editing.
